# Impact of Resistance Training on Bone During 40% Caloric Restriction in Growing Female Rats

**DOI:** 10.1007/s00223-025-01348-y

**Published:** 2025-02-10

**Authors:** Ken D. Sumida, Daniel L. Smithers, Aaron Gerston, Kim A. Lagerborg, S. Victoria Jaque, Fred Caporaso

**Affiliations:** 1 Department of Health Sciences and Food Science Orange, Chapman University, One University Drive, Orange, CA 92866 USA; 2 Department of Kinesiology, California State University, Northridge, Northridge, CA USA

**Keywords:** Diet, Tibia, BMD, Bone mechanical properties

## Abstract

There is a growing trend in the use of severe caloric restrictive diets among normal weight young females that can jeopardize bone health. Using an animal model, the purpose of this study was to determine whether resistance training (RT) could maintain bone health during a 6-week severe caloric restrictive (CR) diet in growing female rats. Twenty-four female rats (~ 8 weeks old) were randomly divided into the following groups: sedentary rats fed a normal diet (*N* = 8), sedentary rats fed a 40% CR diet (*D* = 8), and an RT group fed a 40% CR diet (DT = 8). The DT group climbed a vertical ladder four consecutive times (per exercise session) with weights appended to their tail 3 days/week for a total of 6 weeks. Tibial bone mineral density (BMD) was assessed using dual-energy X-ray absorptiometry scans and bone mechanical properties were measured. After 6 weeks, the body mass (Mean ± SD) of CR-fed groups (*D* & DT = 202.8 ± 10.7 g) was significantly lower than N (275.5 ± 25.3 g). Tibial BMD (g/cm^2^) for D (0.196 ± 0.012) was significantly lower vs. *N* (0.213 ± 0.013), resulting in a 7.9% decline. The tibial BMD for DT (0.206 ± 0.009) resulted in a 3.3% decline compared to N that was not significantly different. Bone mechanical properties were significantly greater for DT compared to D, but not significantly different compared to N. Resistance training has the potential to maintain bone health during severe caloric restriction in growing female rats.

## Introduction

While there are reports examining the positive impact of exercise on bone mineral density during caloric restrictive diets in overweight individuals [1, 2], studies examining the impact of caloric restrictive diets and the potential for physical activity to prevent or mitigate bone loss in non-obese individuals are noticeably scarce. This is especially important given the current media emphasizing physical appearance for women to be thin [3, 4] and the lower bone mass that can result from caloric restrictive diets [5]. The inappropriate perception where being thin is associated with health/beauty has led to the trend for young non-obese females to engage in caloric restrictive diets. In support, the impression of being overweight as undesirable has been reported in female adolescents as early as 4th grade [6]. Adams et al. [7] reported that the perception of an ideal body size as well as opposite gender ideal body type prompted more females than males to engage in weight loss practices as early as the 7th grade. Further, Fayet et al. [8] reported that weight loss regimens were implemented by 43% of college women despite 78% having a healthy body mass index. The highest priority would be to correct the unnecessary implementation of caloric restrictive diets in normal weight individuals due to the association between being thin and health/beauty. In the meantime, if normal weight young females continue to engage in caloric restrictive diets, then understanding the impact of exercise to prevent or mitigate bone loss would be important to maintain bone health and minimize the risk of osteoporosis later in life.

There are challenges when investigating the impact of caloric restrictive diets during the growth period in humans given the numerous confounding variables such as the type of diet employed, stage of maturation, and genetics (to name a few). In contrast, a rat model to investigate the impact of caloric restriction upon the bone has several advantages. First, the ability to employ caloric restrictive diets in animals compared to pair-matched controls can be carefully monitored. Next, given the early sexual maturity and extended time for epiphyseal plates to remain open for growing female rats [9], there is the potential to extrapolate the results to young women and adolescent girls. Finally, in addition to changes in bone mineral density, bone mechanical properties can be measured following diet and/or exercise regimens that cannot be determined in humans. Toward this end, several animal studies have specifically examined the impact of exercise in the prevention of bone loss during significant (i.e., 40% less calories per day) restrictive diets [10–13]. All these studies implemented treadmill running during caloric restriction in female rats and demonstrated that this mode of exercise can mitigate the bone loss associated with dietary restriction [10–13]. Except for one of these prior studies [13], bone mechanical properties were not determined [10–12]. Further, while resistance training has the potential to provide an even greater osteogenic response than treadmill running, implementing this mode of training in animals has been a challenge. However, ladder climbing with weight appended to the animal’s tail has been effective in mimicking resistance training [14] and used extensively in our lab to investigate its impact on bone mineral density [15–20].

To date, no study has examined the impact of resistance training on bone amid significant caloric restriction during the growth period in female animals. Therefore, the purpose of the current study was to determine if a resistance training program, via ladder climbing, could mitigate the bone loss associated with a 40% caloric restrictive diet during the growth period in female rats. We also measured bone mechanical properties in all groups. We hypothesized that resistance training could help maintain bone health resulting in less loss of bone mineral density and less loss of bone mechanical properties during severe caloric restriction.

## Methods

### Animals

The experimental protocol for this study was pre-approved by the Chapman University Institutional Review Board where all applicable institutional and national guidelines for the care of use of animals were followed. Twenty-four female Sprague Dawley rats (initially ~ 180 g, ~ 8 weeks old), obtained from Charles River Laboratories (Wilmington, MA), were housed individually and maintained on a reverse l2/12 h light/dark cycle. The animals were acclimated to their living conditions for 1 week after which they were randomly separated into a sedentary normal fed group (N, *n* = 8), a sedentary caloric restriction group (*D* = 8), and a caloric restriction resistance trained group (DT = 8).

### Caloric Restriction

Animals were pair-fed where a normal fed animal (N) was matched with a corresponding calorie restricted animal (D and DT). The amount of food consumed by the normal fed animal was determined every day at the same time of day (8 a.m.). The matched CR animals were then given 60% of the food eaten by the normal fed animal the preceding day resulting in a 40% caloric restrictive diet. All normal fed animals (N) were allowed free access to food from a diet (Product No. D12450B) provided by Research Diets, Inc. (New Brunswick, NJ). Caloric restrictive groups (D and DT) were fed a modified diet (Product No. D08021202) recommended by Research Diets, Inc. that was supplemented with additional vitamins and minerals so that the only variable was 40% fewer calories (Table 1). Water was provided ad libitum to all animals.

**Table 1:** Diets

Normal diet (D12450B)		Caloric restriction (D08021202):	
Protein, Casein	200.00 g	Protein, Casein	200.00 g
Protein, Cystine	3.00 g	Protein, Cystine	3.00 g
Carbohydrate, Sucrose	345.00 g	Carbohydrate, Sucrose	144.30 g
Carbohydrate, Starch	315.00 g	Carbohydrate, Starch	110.00 g
Carbohydrate, Maltodextrin	35.00 g	Carbohydrate, Maltodextrin	35.00 g
Fiber	50.00 g	Fiber	50.00 g
Fat, Soybean oil	25.00 g	Fat, Soybean oil	25.00 g
Fat, Lard	20.00 g	Fat, Lard	20.00 g
Choline Bitartrate	2.00 g	Choline Bitartrate	2.00 g
Mineral Mix (see below)	50.00 g	Mineral Mix (see below)	50.00 g
Vitamin mix (see below)	10.00 g	Vitamin mix (see below)	10.00 g
Total	1055.00 g	Total	649.30 g

Mineral mix	
Potassium Citrate, Monohydrate	330.00 g
Calcium Phosphate, Dibasic	260.00 g
Calcium Carbonate	110.00 g
Sodium Chloride	51.80 g
Magnesium Sulfate	51.52 g
Magnesium oxide	8.38 g
Ferric Citrate	4.20 g
Manganese Carbonate Hydrate	2.45 g
Zine Carbonate	1.12 g
Chromium Potassium Sulfate	0.39 g
Copper Carbonate	0.21 g
Ammonium Molybdate Tetrahydrate	0.06 g
Sodium Fluoride	0.04 g
Sodium Selenite	0.01 g
Potassium Iodate	0.01 g
Sucrose	179.21 g

Vitamin mix	
Vitamin E Acetate	10.00 g
Niacin	3.00 g
Biotin	2.00 g
Pantothenic Acid	1.60 g
Vitamin D3	1.00 g
Vitamin B12	1.00 g
Vitamin A	0.80 g
Pyridoxine	0.70 g
Riboflavin	0.60 g
Thiamine	0.60 g
Folic acid	0.20 g
Menadione Sodium Bisulfite	0.08 g
Sucrose	978.42 g

*Diets do not contain any soy protein or phytoestrogens

*D08021202: To achieve 40% fewer calories, fed 0.62 g for every gram of D12450B

### Resistance Training

The strength training regimen has previously been described [15–20]. Briefly, the animals were required to climb a vertical ladder with weights (fishing sinkers) appended to their tail. There were 26 rungs across the 1-m ladder. The animals were positioned to ensure that they performed each sequential step where one repetition along the ladder required 26 total lifts by the animal (or 13 lifts per limb). For a given exercise session, the resistance trained animals were operantly conditioned to climb the ladder 4 consecutive times to avoid a vat of water beneath them. The exercised animals trained 3 days/week for a total of 6 weeks. The N and D animals were handled on the same days and times as the DT group to equalize any stress attributable to touching. All animals were weighed at the beginning of the week to monitor weight gains and, for the DT animals, to determine the amount of weight to append to their tails for the remainder of the week. DT animals started with 30% body mass (BM) appended to their tail and each week the amount of weight was elevated by 30% BM until week 5 where they carried 135% of their BM. At week 6, they were carrying 150% of their BM per exercise session.

### Experimental Protocol

To minimize any residual effect of the last training bout, all DT animals were sacrificed 48 h after their final 6-week exercise session along with all sedentary animals. The Flexor Hallucis Longus (FHL) was rapidly dissected from the right hindlimb, weighed, and immediately frozen in liquid nitrogen for the subsequent determination of protein content. The entire left hindlimb was rapidly amputated, positioned, and frozen in liquid nitrogen for the assessment of bone mineral density of the tibia. Prior to the measurement of bone mechanical properties via three-point bending, the tibia from the right hindlimb was dissected and placed in an ethanol/saline (50/50) solution kept at room temperature as described by Turner and Burr [21]. Blood samples were collected, allowed to clot, centrifuged, and the serum was frozen for the subsequent measurement of serum osteocalcin (OC) and pyridinoline (PYD). All tissue and serum samples were kept at − 80ºC until its analysis.

### Chemical Analyses

Protein concentration in the FHL was assessed [22] as an indirect indicator of strength training (i.e., muscle hypertrophy) as reported by Hornberger and Farrar [14] and confirmed in our prior studies [15–20]. A sandwich enzyme-linked immunosorbent assay (ELISA, Biomedical Technologies, Inc., Stoughton, MA) was used to determine serum osteocalcin levels (an indicator of osteoblast activity). The intra-assay and inter-assay variation was < 3%. Serum pyridinoline (an indicator of osteoclast activity) was measured using a competitive enzyme immunoassay (EIA, Quidel Corp., San Diego, CA). The intra-assay variation was < 5% and the inter-assay variation was < 7%. A microplate reader (MaxLine, Molecular Devices Corp., Sunnyvale, CA) was used with the absorbance set at 450 nm for the ELISA and 405 nm for the EIA. A standard curve was generated for all chemical analyses and controls were run to ensure quality. For all standard curves, the Pearson’s Product Correlation Coefficient for linear relationships (i.e., protein), or the Coefficient of Determination for non-linear curves, (i.e., OC and PYD) was greater than 0.99.

### Bone Mineral Density

A Dual-Energy X-ray Absorptiometer (DXA—GE Lunar Prodigy, Chicago, IL) employing the small animal software module (version 6.81) was used to assess the BMD of the entire left tibia. Briefly, the left hindlimb was thawed, positioned, and the entire limb was scanned. Condyle and malleolus curvatures of the tibia were used as anatomical markers to ensure proper positioning and to prevent twisting so that the curvatures were not exaggerated or obliterated. Three consecutive measurements were performed with the hindlimb repositioned between each scan. The reported BMD was the average of three scans, where the coefficient of variation (calculated as [mean/SD] × 100) for repeated scans was 2.04 ± 0.48% (mean ± standard deviation).

### Biomechanical Three-Point Bending Tests

The bone mechanical properties were measured within 1 week of dissection using a three-point bending rig where the tibia was placed onto the stage of a texture analyzer instrument (TA-XT2, Texture Technologies, Ramona, CA). The texture analyzer is an instrument identical in function to an Instron and commonly used in food science. The right tibial bone was removed from the ethanol/saline solution and submerged in saline for 24 h prior to testing at room temperature. The instrument was calibrated using a standard weight and the tibia was patted dry and secured to the rig. The span of the two support points was 14 mm and the deformation rate was 0.9 mm/sec. A medial to lateral force was applied to the midshaft of the bone. The maximal load to failure (Fmax, units = N), energy to failure (EF) determined from the area under the load-deformation curve to the fracture point (units = N x mm), and Stiffness determined from the initial slope (units = N/mm) were assessed using Texture Expert (v. 1.22, Stable Micro Systems Ltd., Surrey, England, UK).

### Calculations and Statistics

Total protein in the FHL was calculated as the product of protein concentration and muscle mass. A one-way ANOVA was employed for all other comparisons, and when a significant F ratio was identified, a Tukey’s post hoc test was used. The level of significance was set at *P* < 0.05 for all statistical comparisons and the results were expressed as the mean ± standard deviation (SD).

## Results

The initial body weight was not significantly different between groups (Table 2). Following the 6-week 40% caloric restrictive diet, the final body weight was significantly lower for D and DT compared to N (Table 2). The FHL mass and protein content were significantly lower for D compared to N but was not significantly different between DT and N (Table 2). To support a resistance training effect, the FHL mass and protein content were significantly higher for DT vs. D (Table 2).

**Table 2:** Body mass and training effect on the flexor hallucis longus

Group	Initial BM (g)	Final BM (g)	FHL Mass (g)	FHL protein (mg protein/muscle)
N	182.4 ± 13.3	275.5 ± 25.3	0.136 ± 0.010	24.1 ± 1.7
D	188.4 ± 10.2	203.3 ± 20.6*	0.116 ± 0.008*	19.2 ± 1.7*
DT	189.4 ± 6.5	202.3 ± 7.5*	0.130 ± 0.014†	22.8 ± 2.0†

*BM* Body mass, *FHL* Flexor Hallucis Longus. *N* Sedentary Normal Fed group (*n* = 8), D = 40% Caloric Restricted Sedentary Group (*n* = 8), DT = 40% Caloric Restricted Resistance Trained Group (*n* = 8). Data are expressed as the mean ± SD.

*Significant difference versus N. †Significant difference versus D

**Fig. 1:**
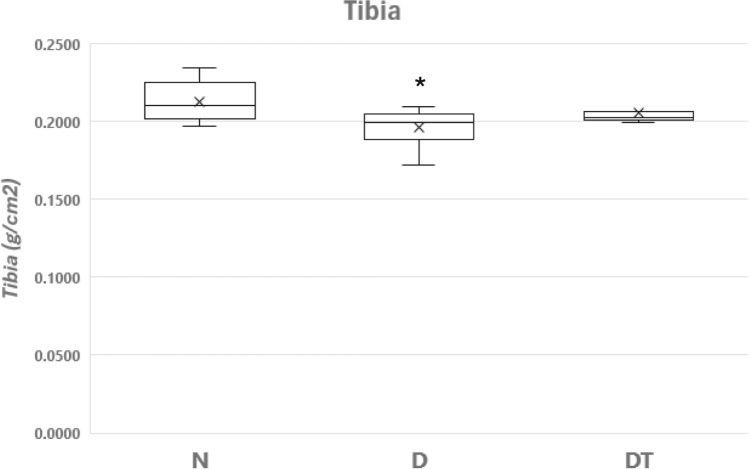
Bone Mineral Density (BMD in g/cm^2^) for the left tibia from the group of normal fed animals (N, *n* = 8), the group fed a 40% caloric restricted diet (D, *n* = 8), and the resistance trained group fed a 40% caloric restricted diet (DT, *n* = 9). Data are expressed as the mean ± SD where the “X” in the box represents the mean and the line represents the median. For DT, to avoid confusion, not depicted is one data point slightly above the box. *Significant difference D vs. N

The bone mineral density from the left tibia was significantly lower for D compared to N resulting in a 7.9% decline (Fig. 1). The DT group exhibited a 3.3% lower BMD compared to N that was not significantly different (*P* = 0.46). Further, BMD was not significantly different (*P* = 0.23) between DT and D (Fig. 1). Concomitant with the decline in BMD was the significantly lower serum OC from D vs. N at the time of sacrifice (Table 3). While OC was lower for DT vs. N, there was no significant difference (*P* = 0.39). In like manner, while the OC was higher for DT vs. D, statistical significance was not achieved (*P* = 0.22). There were no significant differences between groups in serum PYD (Table 3).

**Table 3:** Serum osteocalcin & pyridinoline

Group serum	OC (ng/ml)	Serum PYD (nmol/L)
N	44.77 ± 8.51	3.80 ± 0.67
D	33.14 ± 10.55*	3.93 ± 0.95
DT	39.68 ± 4.61	3.86 ± 0.48

*N* Sedentary Normal Fed Group (*n* = 8), *D* = 40% Caloric Restricted Sedentary Group (*n* = 8), DT = 40% Caloric Restricted Resistance Trained Group (*n* = 8). Data are expressed as the mean ± SD

*Significant difference versus N

**Fig. 2:**
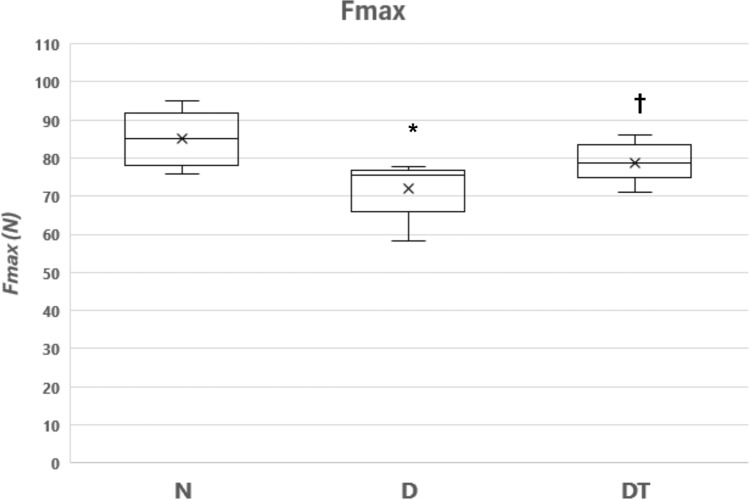
Bone Mechanical Property (Fmax) for the right tibia from the group of normal fed animals (N, *n* = 8), the group fed a 40% caloric restricted diet (D, *n* = 8), and the resistance trained group fed a 40% caloric restricted diet (DT, *n* = 9). Fmax is the amount of force required to break (i.e., fracture) the bone (units = Newtons). Data are expressed as the mean ± SD where the “X” in the box represents the mean and the line represents the median. *Significant difference D vs. N. †Significant difference D vs. DT

**Fig. 3:**
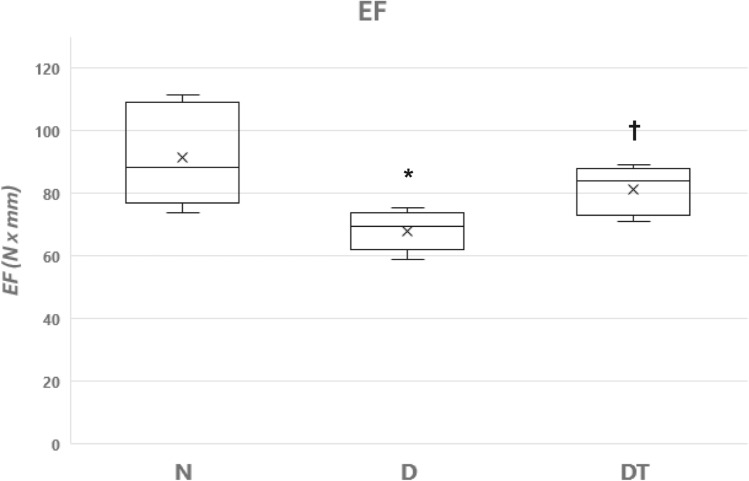
Bone Mechanical Properties (EF, Energy to Failure) for the right tibia from the group of normal fed animals (N, *n* = 8), the group fed a 40% caloric restricted diet (D, *n* = 8), and the resistance trained group fed a 40% caloric restricted diet (DT, n = 9). EF is the Energy to Failure determined from the area under the load-deformation curve to the fracture point (units = Newtons x mm). Data are expressed as the mean ± SD where the “X” in the box represents the mean and the line represents the median. *Significant difference D vs. N. †Significant difference D vs. DT

**Fig. 4:**
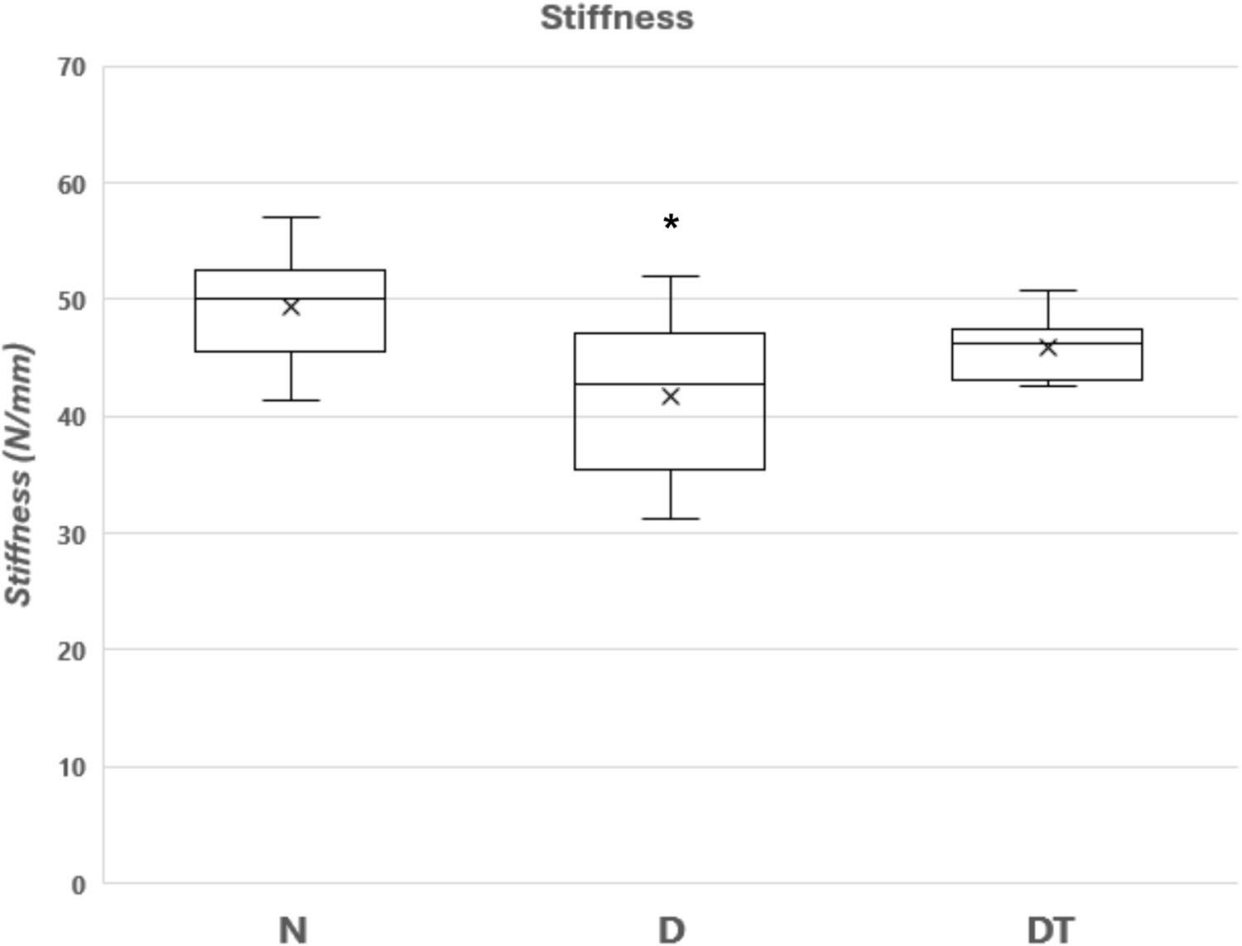
Bone Mechanical Properties (Stiffness) for the right tibia from the group of normal fed animals (N,* n* = 8), the group fed a 40% caloric restricted diet (D,* n* = 8), and the resistance trained group fed a 40% caloric restricted diet (DT, *n* = 9). Stiffness is the slope of the linear portion of the load-deformation curve to the point of fracture (units = Newtons ÷ mm). Data are expressed as the mean ± SD where the “X” in the box represents the mean and the line represents the median. *Significant difference D vs. N

The decline in BMD observed for D resulted in significant reductions in bone mechanical properties (Fmax, EF and Stiffness) of the left tibia compared to N as measured from the three-point bending test (Figs. 2, 3 and 4). While the bone mechanical properties were lower for DT compared to N, there was no significant difference between DT and N for Fmax (*P* = 0.15), EF (*P* = 0.16) and Stiffness (*P* = 0.31). In contrast, bone mechanical properties (Fmax and EF) were significantly lower for D compared to DT (Figs. 2 and 3). While bone stiffness was lower for D vs. DT (Fig. 4), statistical significance was not achieved (*P* = 0.08).

## Discussion

The 40% caloric restrictive diet was supplemented with additional vitamins and minerals where the only variable was the reduction in calories compared to the pair-matched normal fed sedentary group. The reduction in calories for the caloric restricted (D) group resulted in a 7.9% decline in bone mineral density and concomitant declines in bone mechanical properties of the tibia compared to the normal fed group (N). The decline in BMD in the current study is consistent with prior caloric restrictive studies in lean [23] and elderly female rats [24]. In contrast, BMD from the caloric restricted exercised group (DT) resulted in a 3.3% decline in BMD that was not significantly different compared to N. There were no significant BMD differences between DT and N or DT and D. Bone mechanical properties (Fmax and EF) were significantly higher for DT compared to D. While bone mechanical properties were lower for DT compared to N, they were not significantly different. Therefore, the results support our hypothesis that resistance training can mitigate bone loss and maintain bone health during severe caloric restriction.

Diets are typically employed to reverse obesity. However, emphasis on physical appearance and sexual appeal has been associated with being thin and become rampant in television ads, video games, and social media sites [3]. Collectively, the American population has become more self-conscious about body image resulting in the decision to engage in diets to lose weight even when it is not needed to reverse obesity [3]. This is especially apparent in normal weight young females who engage in diets due to body dissatisfaction, insecurity, and/or an inappropriate perception of what constitutes health/beauty [25]. In support, Latimer et al. [26] reported that 28% of American college students were trying to lose weight even though they were under- or at a normal body weight. Wharton et al. [27] reported over 73% of young American women attempted to lose weight even though 78% were normal or underweight. In addition to the media exposure emphasizing thinness, there is scientific interest in understanding the impact of caloric restrictive diets in humans given the reports that diets can extend the lifespan in animals [28]. In particular, the primary purpose of the CALERIE (Comprehensive Assessment of Long-term Effects of Reducing Intake of Energy) trials, supported by the National Institute on Aging, is to determine parallels and/or disparities in humans compared to previous findings in animals. Collectively, the media exposure that emphasizes being thin and the association between caloric restriction and lifespan may prompt a myriad of non-obese individuals to engage in diets [29]. However, the impact of caloric restrictive diets in normal weight individuals has the potential to negatively impact bone health. In support, a report from the CALERIE trials in healthy, non-obese men and women indicated bone loss at osteoporotic fracture sites when they engaged in ~ 25% caloric restrictive diets [5]. In like manner, we observed lower bone mineral densities in the femur from non-obese women who reportedly consumed 55% of their recommended minimum daily requirement [30]. Further, a systematic review of major databases done by Veronese and Reginster [31] revealed a significant reduction in BMD associated with caloric restrictive diets thereby compromising bone health.

A decline in bone health is a natural process of aging where the prevailing bone disease associated with senescence is osteoporosis. Data from the U.S. Census Bureau suggest that the elderly are the fastest growing population in the United States [32]. In the past decade, the 65- and older population grew by 34.2% [32]. Considering the elderly population trends, osteoporosis has the potential to approach epidemic levels [33] where the prevalence of this disease has been known to impact more women than men [34]. Moreover, if caloric restrictive diets are implemented by normal weight young women to obtain a thin body image, the projections for osteoporosis may exceed what is anticipated due to senescence alone. Given the absence of a cure for osteoporosis, prevention becomes of utmost importance where resistance training is an effective method to stimulate an elevation in bone mass that will delay the onset or reduce the severity of bone fractures later in life.

There are numerous reports regarding the use of exercise in the overweight and obese population to attenuate the loss of bone during caloric restrictive diets [1, 2, 35]. However, human studies specifically examining the impact of exercise to prevent or mitigate bone loss associated with caloric restrictive diets in normal weight individuals are uncommon. This is most likely due to the challenge in human data collection and/or prior animal studies that have shed light on the outcome minimizing the need to engage in numerous human studies. In support, Bloomfield’s lab reported that during a 40% energy restrictive diet, treadmill running 3–4 times per week offered a partial protection from tibial bone loss in female rats [10–12]. In another well-controlled study employing treadmill exercise during moderate caloric restriction (20% overall less energy) and severe caloric restriction (40% overall less energy) [13], they reported few adverse effects on the femur with the moderate caloric restriction combined with exercise. Meanwhile, the combined impact of exercise during severe caloric restriction mitigated the detrimental effects on bone compared to caloric restriction in the absence of exercise [13]. In this study, bone mechanical properties were assessed where severe energy restricted exercised female rats demonstrated a trend toward greater structural properties of the femur compared to severe energy restricted controls [13]. The results of the current study support all their treadmill findings [10–13] and demonstrate that resistance training can also attenuate bone loss in the tibia associated with a 40% caloric restrictive diet, but we also note some differences. The treadmill running studies employed a 12-week caloric restrictive diet and noted improved structural tibial bone properties [10–12] and elevated bone mechanical properties of the femur [13]. In contrast, we administered a 40% caloric restrictive diet for 6 weeks where our resistance training protocol resulted in improvements in tibia BMD and significant elevations in bone mechanical properties (i.e., Fmax and EF) for the DT group compared to the pair-matched D group. Therefore, the more rapid training-induced alterations in BMD support the enhanced effectiveness of strength exercise during caloric restriction. Further, the enhanced tibial bone mechanical properties for DT vs. D confirm the improved structural bone properties noted by prior exercise studies [11, 12, 36] and the elevations in bone mechanical properties observed in the femur [13]. In opposition to the current results and prior treadmill studies in rats, McGrath et al. [37] implemented a 30% caloric restrictive diet in female mice and reported that 6 weeks of chronic exercise had a negative impact on the femur. They implemented voluntary wheel running where the caloric restricted animals averaged 10 km/day [37]. In contrast, the forced treadmill running employed by others [10–13] required rats to run 3–4 days/week and the distance covered per exercise session was 4 to 8 times lower than the mice reported by McGrath et al. [37]. Thus, it is possible that more prolonged and/or daily aerobic exercise during caloric restriction, as observed by McGrath et al. [37], can eventually yield detrimental effects on the bone. Next, Hattori et al. [38] examined the impact of voluntary wheel running combined with a 30% caloric restrictive diet in adult male rats and found no changes in bone loss, bone morphology or bone strength of the tibia compared to sedentary controls [38]. We have no explanation for the discrepancy among the various caloric restrictive diet studies in animals. However, we recognize the potential for sex differences and/or the use of prolonged daily running as the mode of exercise compared to treadmill exercise or resistance training three times per week.

Despite the disparities from prior animal studies pertaining to running as the mode of exercise, the strength training-induced maintenance of BMD for DT compared to N is consistent with our prior reports on the effectiveness of this mode of exercise in promoting an osteogenic response in maturating animals [15–20]. Further, the current report confirms previous findings in humans [39–41] and animals [15–20, 42, 43] pertaining to higher osteoblast activity as the potential mechanism for the reported maintenance of BMD as supported by the serum OC from the DT and N groups. Accordingly, the lower serum OC for D vs. N also supports the potential mechanism for the reductions in BMD attributable to caloric restriction, i.e., a reduction in osteoblast activity. Last, our results regarding training-induced elevations in bone mechanical properties of the tibia are also consistent with our prior reports [15–19] and others that have examined the femur after treadmill running [13], jumping exercise [36], and tower climbing [44] in normal fed rats vs. sedentary controls. Therefore, the elevation in bone mechanical properties for DT is consistent with the higher BMD compared to D. Collectively, the current results emphasize the importance of incorporating resistance training to maintain bone health during severe caloric restriction.

Finally, we recognize several limitations of this study. First, given the impact of hormones upon the bone, it would have been prudent to measure insulin-like growth factor, estrogen, parathyroid hormone, and/or leptin. Next, assessing BMD and serum markers for all animals prior to the implementation of the study would have allowed for a longitudinal assessment. As it pertains to BMD, the DXA measures mass per area where differences in bone size can result in misinterpretations with the use of DXA [45]. In addition, DXA does not provide information regarding bone microarchitecture. As such, we acknowledge that having access to micro-CT (micro-computed tomography) or pQCT (peripheral quantitative computed tomography) as well as histomorphometry data and/or employing tartrate-resistant acid phosphatase to assess osteoclast number would have added to our interpretation of the results. We also recognize that bones were stored in an ethanol/saline solution at room temperature where potential changes in the organic matrix due to the ethanol as well as potential denaturation of proteins were possible that could alter the mechanical testing results. However, the tibia was treated in the same manner for all groups prior to bone testing that allows for relative comparisons. Despite all these limitations, the bone mechanical properties supported the alterations in bone mineral density for a given group. Further we acknowledge the small sample size for each group that may have prevented achieving statistically significant differences between groups for several measurements. Given the limited amount that is awarded for internal grants, we were confined to focus on the major factors after the experimentation that would either support or refute our hypothesis. Last, our study employed 6 weeks of caloric restriction. Thus, it is possible that strength training during more prolonged caloric restriction may yield different results.

## Conclusions

To the extent that the current results in growing female rats can be extrapolated to humans, when significant caloric restrictive diets are employed by normal weight young females, incorporating a resistance training regimen is essential. A strength training program can help to mitigate the bone loss associated with severe caloric restriction and assist in maintaining bone health thereby minimizing the onset or reducing the severity of osteoporosis in old age. We acknowledge that further investigations are warranted to determine possible sex differences on the impact of resistance training on bone during severe caloric restrictive diets as well as its impact on bone during modest caloric restrictive diets (e.g., ~ 25%).
